# Tailoring the Oxygen Content of Graphite and Reduced Graphene Oxide for Specific Applications

**DOI:** 10.1038/srep21715

**Published:** 2016-02-25

**Authors:** Naoki Morimoto, Takuya Kubo, Yuta Nishina

**Affiliations:** 1 Graduate School of Medicine, Dentistry, and Pharmaceutical Sciences, Division of Pharmaceutical Sciences, Okayama University, Tsushimanaka, Kita-ku, Okayama 700-8530, Japan; 2 Graduate School of Natural Science and Technology, Okayama University, Tsushimanaka, Kita-ku, Okayama 700-8530, Japan; 3 Precursory Research for Embryonic Science and Technology, Japan Science and Technology Agency, 4-1-8 Honcho, Kawaguchi, Saitama 332-0012, Japan; 4 Research Core for Interdisciplinary Sciences, Okayama University, Tsushimanaka, Kita-ku, Okayama 700-8530, Japan

## Abstract

Graphene oxide (GO) is widely recognized as a promising material in a variety of fields, but its structure and composition has yet to be fully controlled. We have developed general strategies to control the oxidation degree of graphene-like materials via two methods: oxidation of graphite by KMnO_4_ in H_2_SO_4_ (oGO), and reduction of highly oxidized GO by hydrazine (rGO). Even though the oxygen content may be the same, oGO and rGO have different properties, for example the adsorption ability, oxidation ability, and electron conductivity. These differences in property arise from the difference in the underlying graphitic structure and the type of defect present. Our results can be used as a guideline for the production of tailor-made graphitic carbons. As an example, we show that rGO with 23.1 wt% oxygen showed the best performance as an electrode of an electric double-layer capacitor.

Controlling the oxygen content in graphite- and graphene-like materials is important for tailoring the functionalities appropriate to their application in areas such as optoelectronics, and physical-, biological-, and energy-related materials^1^,^2^,^3^,^4^. Graphene oxide (GO) containing a low quantity of carboxyl and carbonyl groups will be suitable for the production of highly conductive and defect-free graphene-like materials via reduction (Fig. 1a). In contrast, oxygen-containing functional groups are necessary for the production of functionalized GO to form composites with metals^5^,^6^,^7^,^8^ and organic molecules^9^,^10^,^11^,^12^,^13^, and for doping with heteroatoms^14^,^15^ (Fig. 1b). Therefore, fine tuning the graphene structure is important for performance optimization. When graphite is partially oxidized, complete exfoliation does not occur and graphite oxide (GtO) is obtained rather than GO^16^,^17^. Although GO sometimes shows superior performance, GtO sometimes performs better when high electronic conductivity and/or an assembled structure are required^18^,^19^,^20^. In this paper, we show how to control the oxygen content of graphitic carbons by the oxidation of graphite and by the reduction of highly oxidized GO, and propose guidelines for tailor-making graphene-like materials for applications.

Various GO structures have been reported in the literature, for example, the interlayer spacing can vary from 0.6 to 1.0 nm^21^,^22^, various oxygen functionalities can be present^23^, and the domain size can range from several nanometers to 200 μm^24^, depending on the synthetic procedure. Control of the oxygen content and oxygen-containing functional group distribution can be achieved by tuning the oxidation conditions of graphite^25^,^26^,^27^,^28^,^29^,^30^,^31^,^32^,^33^,^34^,^35^ or the reduction conditions of GO^36^,^37^,^38^,^39^. However, fine tuning of the GO structure and the relationship between the oxygen content and GO properties have not been investigated.

## Results and Discussion

**Figure 2:**
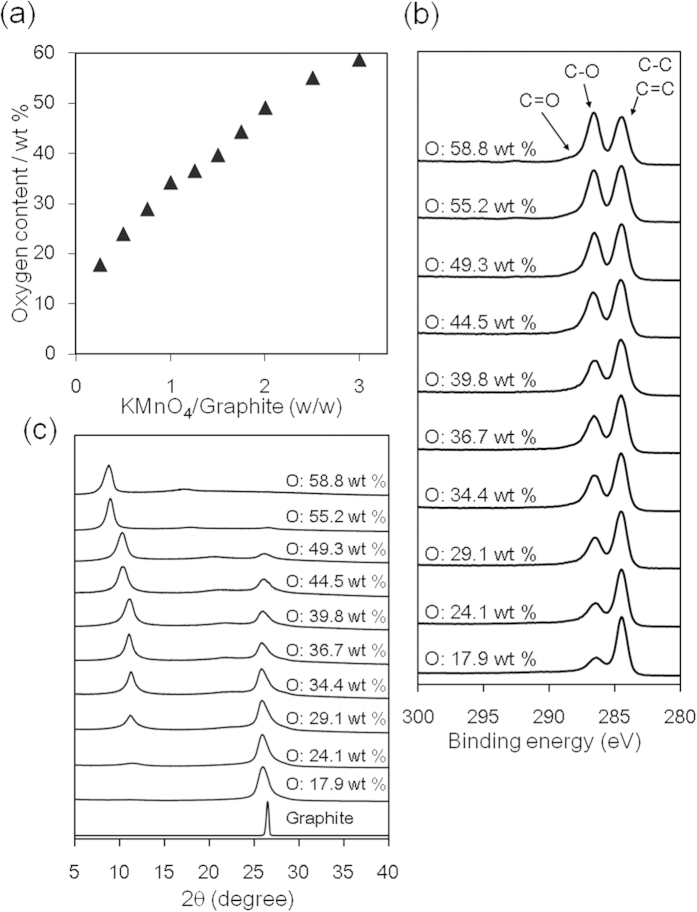
Characterization of oGO. (**a**) Oxygen content determined by elemental analysis, (**b**) XPS, and (**c**) XRD spectra of oGO with different oxygen contents.

GO is generally prepared by treating graphite with three times its mass of KMnO_4_ in H_2_SO_4_^40^. Here, we refer to an oxidized graphite where the oxygen content was controlled by the oxidation step as oGO. If the amount of KMnO_4_ is decreased, a heterogeneous mixture of GO and unoxidized graphite is formed. We attempted to suppress the localized oxidation of graphite by optimizing the oxidation conditions to form a uniform graphite intercalation compound (GIC) before quenching with H_2_O and H_2_O_2_ (See ESI)^41^. The disappearance of the graphite (002) peak, monitored by X-ray diffraction (XRD), suggests the formation of GIC *in situ* (Fig. S1). The oxygen content was evaluated by CHN elemental analysis, as shown in Fig. 2a. As the mass ratio of KMnO_4_/graphite was decreased from 3.0 to 0.25, the oGO oxygen content decreased from 59 to 18 wt%. In the X-ray photoelectron spectroscopy (XPS) analysis, the peak intensity at 287 eV, which is assigned to C–O bonds, decreased as the amount of KMnO_4_ decreased (Fig. 2b). The XRD patterns showed that the graphitic structure remained until the oxygen content of oGO exceeded ~50 wt% (Fig. 2c). This means that completely exfoliated single-layer oGO requires an oxygen content greater than 50 wt%.

**Figure 3:**
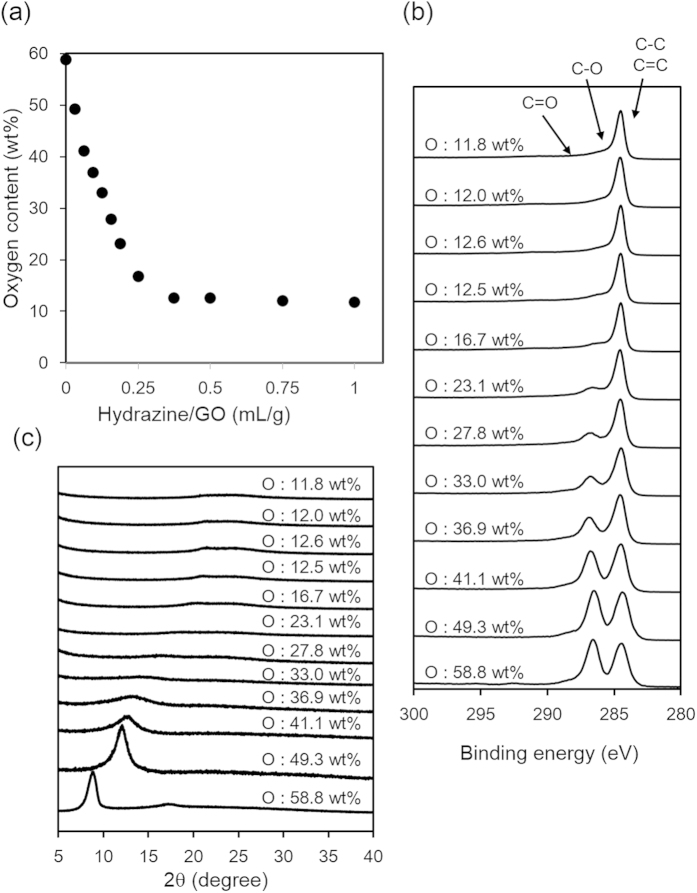
Characterization of rGO. (**a**) Oxygen content determined by elemental analysis, (**b**) XPS, and (**c**) XRD spectra of rGO with different oxygen contents.

We then attempted to control the oxidation degree by the reduction of highly oxidized oGO (58.8 wt% O), which was synthesized using 3.0 weight equiv. of KMnO_4_^42^,^43^. Here, we refer to the reduced GO as rGO. oGO (2.0 g, 58.8 wt% O) was dispersed in water (200 mL), and then hydrazine hydrate was added, before heating at 90 °C for 2 h^44^. After cooling, the product was washed three times by centrifugation with water, and then dried under vacuum at 50 °C for 10 h. The peaks of the UV-Vis spectra of the rGO dispersions gradually shifted to longer wavelength as the amount of hydrazine was increased, suggesting that progressive reduction of oGO occurred (Fig. S2). Elemental analysis of the rGO showed that there was a clear inverse correlation between the amount of hydrazine and the oxygen content (Fig. 3a). When the ratio of hydrazine/GO (mL/g) was increased from 0.03 to 0.25, the oxygen content of rGO proportionally decreased from 48 to 17 wt%. The oxygen content gradually decreased until the hydrazine/GO ratio reached 0.3 and, further reduction did not occur: the oxygen content did not decrease to less than 11 wt% with this reduction method^42^,^43^. The ratio of the intensity of the D-band relative to intensity of the G-band (D/G ratio) of the Raman spectra did not significantly change after reduction with hydrazine, suggesting incomplete recovery of the graphene structure (Fig. S3). The C 1s XPS of rGO with different oxygen contents was recorded so that the the oxygen-containing functional group distributions could be identified (Fig. 3b). The peak at 287 (C–O bond) decreased as the oxygen content decreased (Fig. S4). In the XRD spectra, a high angle peak and a decrease of the GO peak was observed as the reduction proceeded, and this peak disappeared when the oxygen content was less than 33 wt%. No peak appeared at 2θ = 25°–27°, indicating that the layered graphitic structure was not recovered (Fig. 3c).

The correlations between the oxygen content and the properties of oGO and rGO are important benchmarks when utilising GO in practical applications. Here we have investigated methylene blue (MB) adsorption, metal adsorption, the oxidation ability, and the electrical conductivity of oGO and rGO with various oxygen contents, prepared under different oxidation/reduction conditions.

**Figure 4:**
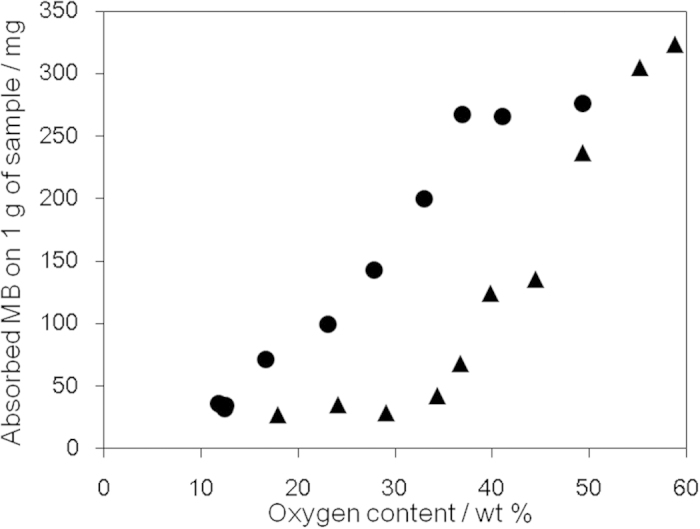
Methylene blue adsorption on oGO and rGO with different oxygen contents: oGO (▴) and rGO (●).

Adsorption of MB is used to evaluate the surface area of graphitic materials, with 1 mg of adsorbed MB equating to a surface area of 2.54 m^2  ^45. oGO and rGO were treated with a known amount of MB solution, and the suspended oGO and rGO were removed by filtration using a membrane filter. The adsorbed amount of MB was calculated by the absorbance at 298 nm (Fig. 4). As the oxygen content of oGO (▴ in Fig. 4) increased, the adsorption of MB gradually increased, presumably because of the progress of exfoliation, which is supported by XRD analysis (Fig. 3c). rGO (● in Fig. 4) always showed higher adsorption ability than oGO, presumably because the precursor oGO (58.8 wt% O) used for the preparation of rGO was completely exfoliated. Interestingly, MB adsorption on rGO with an oxygen content of 50–35 wt% did not change, which suggests that the single-layer structure is retained over this composition range. However, MB adsorption gradually decreased as the oxygen content was further decreased presumably because of the π–π stacking of rGO sheets.

**Figure 5:**
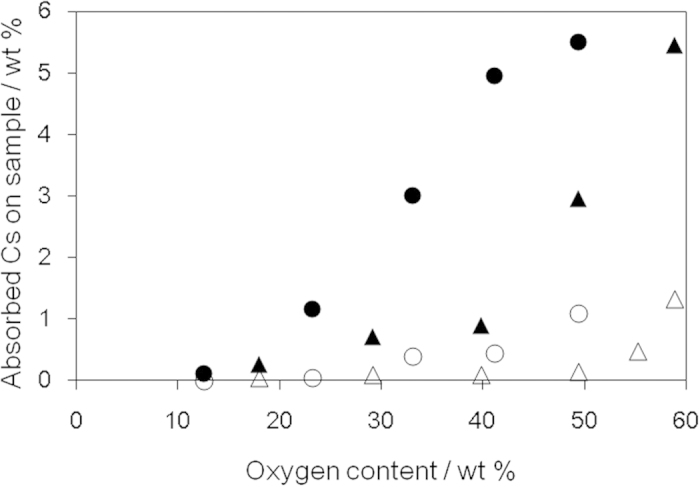
Cs adsorption on oGO and rGO with different oxygen contents. CsCl (oGO (△), rGO (●)) and Cs_2_CO_3_ (oGO (▴), rGO (●)) were used as the cesium sources.

A promising application of GO is to remove poisonous materials, with the oxygen-containing functional groups and defects on the GO surface providing metal capturing sites^46^. The effect of oxygen-containing functional groups on the adsorption of metal ions was investigated using oGO and rGO. In this study, we focused on cesium, because one of the radioisotopes of cesium has relatively long half-life time, high volatility, high activity, and poses considerable ecological problems^47^,^48^. Aqueous solutions of neutral and basic cesium salts (CsCl and Cs_2_CO_3_) were added to oGO and rGO dispersions, and the amount adsorbed measured by atomic absorption analysis (Fig. 5). Cs_2_CO_3_ was more readily adsorbed than CsCl, which suggests that acidic oxygen-containing functional groups contribute to the adsorption via ionic interactions. The adsorbed cesium content increased as the oxygen content of oGO and rGO increased. rGO showed a higher adsorption ability than oGO, similar to the MB adsorption (Fig. 4), especially when the oxygen content was higher than 40 wt%.

**Figure 6:**
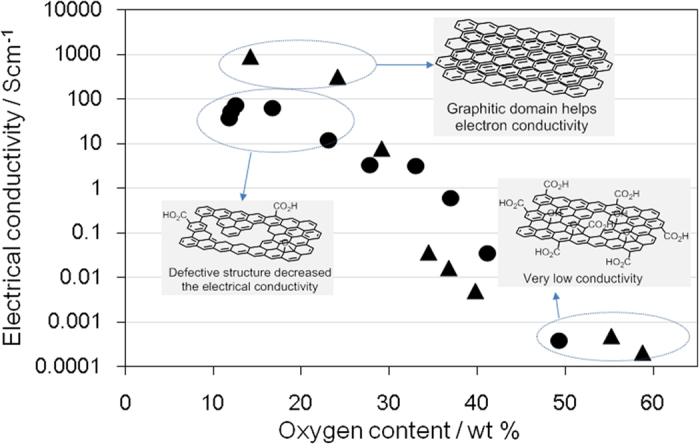
Electrical conductivity of oGO and rGO with different oxygen contents: oGO (▴) and rGO (●).

Electron conductivity is another of the important properties of graphene-like materials. To evaluate the electron conductivity, rGO and oGO were pelletized before four-point probe measurement. The electrical conductivity of oGO dramatically decreased when the oxygen content exceeded ca. 25 wt% (Fig. 6). Combining the XRD spectra (Fig. 3c) with Fig. 6 suggests that the appearance of the XRD peak at around 10°, assigned as GO, and decrease of the graphite (002) peak at 26° dramatically suppressed the electrical conductivity of oGO. For a medium oxygen content (ca. 30–40 wt%), rGO showed better conductivity than oGO presumably because insulating GO was not present, as shown in the XRD spectra (Fig. 3c). By contrast, for a low oxygen content (ca. 10–20 wt%), the conductivity of rGO was less than that of oGO. Incomplete recovery of the sp^2^ domains would inhibit the electron conductivity of rGO, because the original oGO used for the production of rGO was highly oxidized (58.8 wt% O) and therefore contained many defects. To further investigate the influence of the original defects, each oGO was reduced using an excess amount of hydrazine (roGO) for the electron conductivity measurement. As the oxygen content of the original oGO increases, the electrical conductivity of the roGO decreases (Table 1). These results suggest that irreversible damage of the graphene structure of oGO occurrs as the level of oxidation increases.

**Figure 7:**
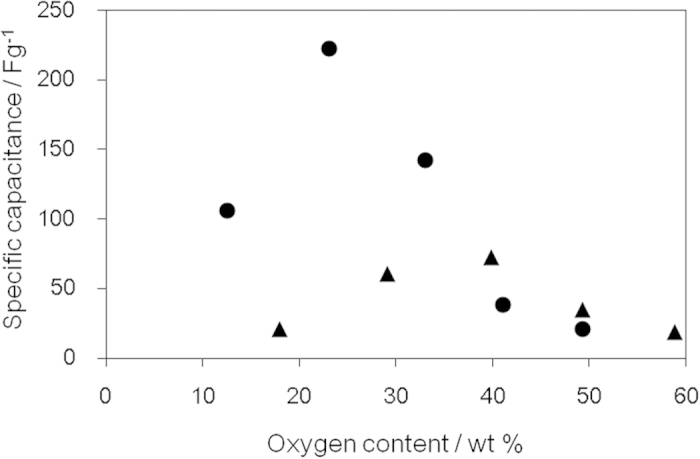
Specific capacity measurement using oGO and rGO with different oxygen content determined by cyclic voltammogram; oGO (▴) and rGO (●). Cyclic voltammetry was performed at a scan rate of 20 mVs^−1^.

The surface area and electrical conductivity of a material are closely related to the capacitance, when that material is used as the electrodes of electric double-layer capacitors^49^,^50^,^51^,^52^. Oxygen-containing functional groups on GO can also contribute to an increase in the capacitance by electrochemical redox reactions. However, as the oxygen content increases, the electron conductivity decreases. Therefore, there must be an optimum oxygen content to obtain the maximum capacitance. The specific capacitance of oGO and rGO was measured with cyclic voltammetry and rGO (23.1 wt% O) showed the highest capacitance (Fig. 7). This maximum must be derived from the balance of high surface area (Fig. 4) and high electron conductivity (Fig. 6).

**Figure 8:**
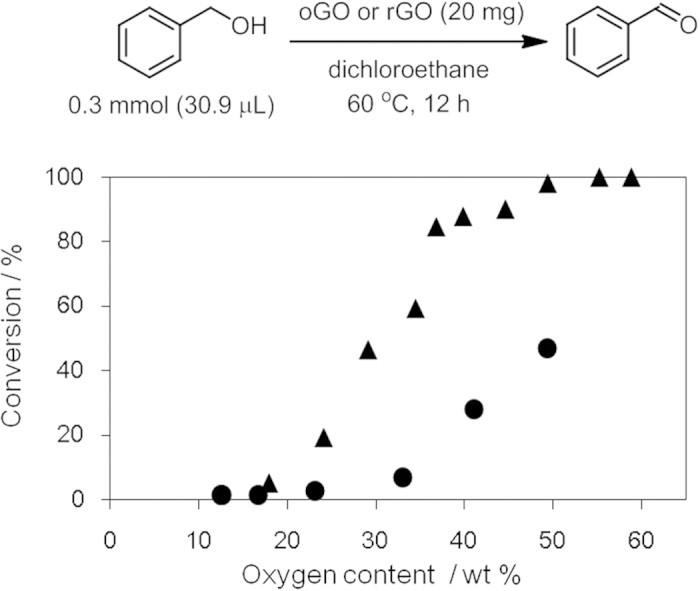
Oxidation of benzylalcohol by GO with different oxygen contents; oGO (▴) and rGO (●).

GO is reduced by metals, alcohols, and microorganisms^42^,^43^. In other words, GO can function as an oxidant. When applying GO to composites and devices, oxidation of other materials, such as metals and polymers, might disrupt the performance. Furthermore, oxidative stress is not advisable for body tissues when GO is used for biological applications. However, a method to evaluate the oxidation capability of GO has not yet been developed. Here we focused on the transformation of benzyl alcohol to benzaldehyde in an inert atmosphere to evaluate the oxidation capability of oGO and rGO^53^. oGO and rGO with higher oxygen contents showed higher conversion of benzyl alcohol (Fig. 8). However, rGO showed a lower oxidation capability than oGO with the same oxygen content. This suggests that the oxygen- containing functional groups have different oxidizing capabilities, and implies that the more oxidizing functional groups of rGO were easily removed by hydrazine. Therefore, pre-reduction of oGO with hydrazine would be an effective way to prevent the unwanted oxidation of the materials that contact with oGO.

## Conclusions

We have achieved control of the degree of oxidation of oGO and rGO via the oxidation of graphite with KMnO_4_ or the reduction of highly oxidized oGO with hydrazine in ca. 5 wt% steps. Even though the oGO and rGO may contain the same amount of oxygen, the properties, such as adsorption ability, electrical conductivity, and oxidation ability, varied depending on the preparation method. Because of its high surface area, moderate conductivity, and low oxidizing ability, we are currently focusing on rGO with 20–40 wt% oxygen content for practical application such as electric double-layer capacitors, thermoconductive films, reinforcing fillers of polymers, support materials of catalysts, and biosensors.

## Methods

### Materials

Graphite (SP-1) was purchased from BAY CARBON Inc. KMnO_4_, H_2_SO_4_, H_2_O_2,_ Cs_2_CO_3_, hydrazine hydrate, and K_2_Cr_2_O_7_ were purchased from Wako Pure Chemical Industries, Ltd. Benzylalcohol and 1,2-dichloroethane were purchased from Tokyo Chemical Industry Co. All reagents were used directly without further purification.

### Apparatus

Gas chromatography (GC) analysis was performed on Shimadzu GC-2014 equipped with FID detector. Fourier transform infrared (FT-IR) was measured by JASCO ATR PRO450-S with Ge. X-ray diffraction (XRD) was measured by PANalytical Co. X’ part PRO using Cu Kα radiation (λ = 1.541 Å) in the 2θ range of 2–75°. The operating tube current and voltage were 40 mA and 40 kV, respectively. The data was collected at the step size of 0.017° and the type of scan was continuous. Energy dispersive X-ray spectroscopy (EDX) was measured by SHIMADZU. Co. Rayny EDX-700HS. The operating tube current and voltage were 100 μA and 15 kV, respectively. Rh was used as X-ray tube. Solid state^13^ C NMR spectra were obtained by JEOL JNM-ECA400. Scanning electron microscopy (SEM) was performed on the JEOL JSM-6701F scanning electron microscope operating at 5 kV. Freeze-dried of GO was made by using an ADVANTEC DRZ350WC. Electrical conductivity was measured by using MITSUBISHI CHEMICAL ANALYTECH MCP-T610.

### Oxidation of graphite

Graphite (SP-1, Bay Carbon Inc.; 3.0 g) was added to H_2_SO_4_ (75 mL) and then the designated amount of KMnO_4_ was slowly added at 10 °C with stirring at 200 rpm. The mixture was kept at 35 °C for 2 h before quenching with H_2_O (75 mL) under vigorous stirring and cooling so that temperature does not exceed 50 °C. H_2_O_2_ (30%, 7.5 mL) was slowly added, with continuous stirring, for 30 min at ambient temperature. The reaction mixture was purified by centrifugation.

### Reduction of GO

2.0 g of oGO (58.8 wt% O) was diluted in 200 mL of water, and designated amount of hydrazine was added. The mixture was heated at 90 °C, then filtered and washed by water. The solid product was recovered and freeze-dried for characterization.

### Oxidation of benzyl alcohol

Benzyl alcohol 0.3 mmol) was added to a mixture of oGO or rGO (20 mg) and 1,2-dichloroethane (0.5 mL), and the mixture was heated at 60 °C for 12 h. The reaction mixture was analyzed by gas chromatography using dodecane as an internal standard.

## Additional Information

**How to cite this article**: Morimoto, N. *et al*. Tailoring the Oxygen Content of Graphite and Reduced Graphene Oxide for Specific Applications. *Sci. Rep*. **6**, 21715; doi: 10.1038/srep21715 (2016).

## Supplementary Material

Supplementary Information

## Figures and Tables

**Figure 1:**
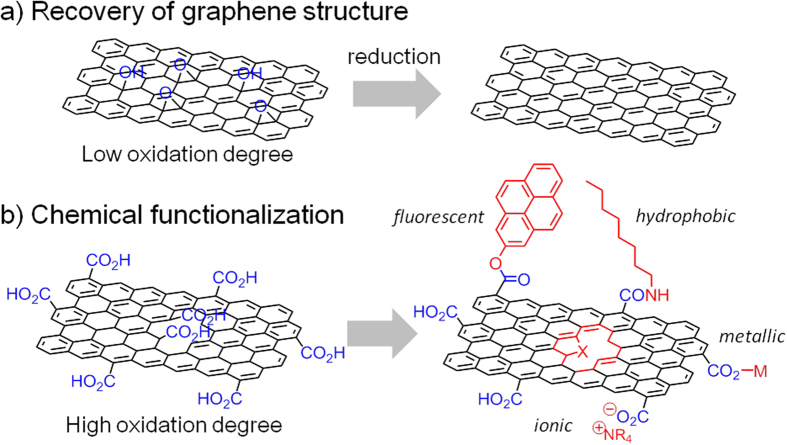
Functionalization of GO with different degrees of oxidation.

**Table 1:**
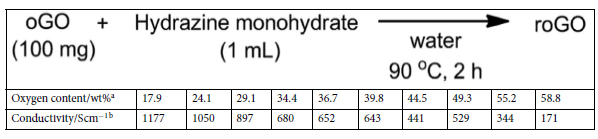
Electrical conductivity of roGO produced by the reduction of different oGO using an excess amount of hydrazine.

^a^Oxygen content of original oGO. ^b^Electrical conductivity of roGO.

## References

[b1] Chen D., Feng H. & Li J. Graphene oxide: preparation, functionalization, and electrochemical applications. Chem. Rev. 112, 6027–6053 (2012). 22889102 10.1021/cr300115g

[b2] Zhu Y., James D. K. & Tour J. M. New routes to graphene, graphene oxide and their related applications. Adv. Mater. 24, 4924–4955 (2012). 22903803 10.1002/adma.201202321

[b3] Huang C., Li C. & Shi G. Graphene based catalysts. Energy Environ. Sci. 5, 8848–8868 (2012).

[b4] Ruoff R. Graphene Calling all chemists. Nature Nanotecnol. 3, 10–11 (2008). 10.1038/nnano.2007.432 18654440

[b5] Bai S. & Shen X. Graphene–inorganic nanocomposites. RSC Adv. 2, 64–98 (2012).

[b6] Yamamoto S., Kinoshita H., Hashimoto H. & Nishina Y. Facile preparation of Pd nanoparticles supported on single-layer graphene oxide and application for the Suzuki–Miyaura cross-coupling reaction. Nanoscale 6, 6501–6505 (2014). 24838740 10.1039/c4nr00715h

[b7] Huang X., Qi X., Boey F. & Zhang H. Graphene-based composites. Chem. Soc. Rev. 41, 666–686 (2012). 21796314 10.1039/c1cs15078b

[b8] Goncalves G. . Surface modification of graphene nanosheets with gold nanoparticles: The role of oxygen moieties at graphene surface on gold nucleation and growth. Chem. Mater. 21, 4796–4802 (2009).

[b9] Kuilla T. . Recent advances in graphene based polymer composites. Prog Polym. Sci. 35, 1350–1375 (2010).

[b10] Kim H., Abdala A. A. & Macosko C. W. Graphene/polymer nanocomposites. Macromolecules 43, 6515–6530 (2010).

[b11] Stankovich S., Piner R. D., Nguyen S. T. & Ruoff R. S. Synthesis and exfoliation of isocyanate-treated graphene oxide nanoplatelets. Carbon 44, 3342–3347 (2006).

[b12] Georgakilas V. . Functionalization of graphene: covalent and non-covalent approaches, derivatives and applications. Chem. Rev. 112, 6156–6214 (2012). 23009634 10.1021/cr3000412

[b13] Eigler S. & Hirsch A. Chemistry with graphene and graphene oxide—challenges for synthetic chemists. Angew. Chem. Int. Ed. 53, 7720–7738 (2014). 10.1002/anie.201402780 24962439

[b14] Panchakarla L. S. . Synthesis, structure, and properties of boron- and nitrogen-doped graphene. Adv. Mater. 21, 4726–4730 (2009).

[b15] Choi C. H. . S. I. B, N- and P, N-doped graphene as highly active catalysts for oxygen reduction reactions in acidic media. J. Mater. Chem. A 1, 3694–3699 (2013).

[b16] Wick P. . Classification framework for graphene-based materials. Angew. Chem. Int. Ed. 53, 7714–7718 (2014). 10.1002/anie.201403335 24917379

[b17] Bianco A. . All in the graphene family – A recommended nomenclature for two-dimensional carbon materials. Carbon 65, 1–6 (2013).

[b18] Pan D. . Li storage properties of disordered graphene nanosheets. Chem. Mater. 21, 3136–3142 (2009).

[b19] Nilsson J., Neto A. H. C., Guinea F. & Peres N. M. R. Electronic properties of graphene multilayers. Phys. Rev. Lett. 97, 266801 (2006). 17280447 10.1103/PhysRevLett.97.266801

[b20] Yoo J. J. . P. M. Ultrathin planar graphene supercapacitors. Nano Lett. 11, 1423–1427 (2011). 21381713 10.1021/nl200225j

[b21] Si Y., Samulski & Synthesis of water soluble graphene. Nano Lett. 8, 1679–1682 (2008). 18498200 10.1021/nl080604h

[b22] Mkhoyan K. A. . Evidence of graphitic AB stacking order of graphite oxides. Nano Lett. 9, 1058–1063 (2009).19199476

[b23] Dreyer D. R., Park S., Bielawski C. W. & Ruoff R. S. The chemistry of graphene oxide. Chem. Soc. Rev. 39, 228–240 (2010). 20023850 10.1039/b917103g

[b24] Zhou X. & Liu Z. A scalable, solution-phase processing route to graphene oxide and graphene ultralarge sheets. Chem. Commun. 46, 2611–2613 (2010). 10.1039/b914412a 20449324

[b25] Wang X. . Correlation between the adsorption ability and reduction degree of graphene oxide and tuning of adsorption of phenolic compounds. Carbon 9, 101–112 (2014).

[b26] Lee D. W. & Seo J. W. sp^2^/sp^3^ carbon ratio in graphite oxide with different preparation times. J. Phys. Chem. C. 115, 2705–2708 (2011).

[b27] Krishnamoorthy K., Veerapandian M., Yun K. & Kim S.-J. The chemical and structural analysis of graphene oxide with different degrees of oxidation. Carbon 53, 38–49 (2013).

[b28] Wang G., Sun X., Liu C. & Lian J. Tailoring oxidation degrees of graphene oxide by simple chemical reactions. Appl. Phys. Lett. 99, 053114 (2011).

[b29] Kadam M. M. . Impact of the degree of functionalization of graphene oxide on the electrochemical charge storage property and metal ion adsorption. RSC Adv. 4, 62737–62745 (2014).

[b30] Lee W., Suzuki S. & Miyayama M. Lithium storage properties of graphene sheets derived from graphite oxides with different oxidation degree. Ceram. Int. 39, S753–S756 (2013).

[b31] Thangavel S. & Venugopal G. Understanding the adsorption property of graphene-oxide with different degrees of oxidation levels. Powder Technol. 257, 141–148 (2014).

[b32] Zhao L. . Enhancement on the permeation performance of polyimide mixed matrix membranes by incorporation of graphene oxide with different oxidation degrees. Polym. Adv. Technol. 26, 330–337 (2015).

[b33] Krishnamoorthy K., Veerapandian M., Yun K. & Kim S. J. The chemical and structural analysis of graphene oxide with different degrees of oxidation. Carbon 53, 38–49 (2013).

[b34] Wu R. . Control of the oxidation level of graphene oxide for high efficiency polymer solar cells. RSC Adv. 5, 49182–49187 (2015).

[b35] Zhang G. . The surface analytical characterization of carbon fibers functionalized by H_2_SO_4_/HNO_3_ treatment. CARBON 46, 196–205 (2015).

[b36] Wang X. . H. Correlation between the adsorption ability and reduction degree of graphene oxide and tuning of adsorption of phenolic compounds. Carbon 69, 101–112 (2014).

[b37] Zhang W. . Insight into the capacitive properties of reduced graphene oxide. ACS Appl. Mater. Interfaces 6, 2248–2254 (2014). 24456342 10.1021/am4057562

[b38] Song N. . Thermally reduced graphene oxide films as flexible lateral heat spreaders. J. Mater. Chem. A 2, 16563–16568 (2014).

[b39] Chien C.-T. . Tunable photoluminescence from graphene oxide. Angew. Chem. Int. Ed. 51, 6662–6666 (2012). 10.1002/anie.201200474 22623281

[b40] Hummers W. S. & Offeman R. E. Preparation of graphitic oxide. J. Am. Chem. Soc. 80, 1339 (1958).

[b41] Nakajima T., Mabuchi A., Hagiwara R. & Watanabe N. Discharge characteristics of graphite fluoride prepared via graphite oxide. J. Electrochem. Soc. 20, 93–98 (1987).

[b42] Chua C. K. & Pumera M. Chemical reduction of graphene oxide: a synthetic chemistry viewpoint. Chem. Soc. Rev. 43, 291–312 (2014). 24121318 10.1039/c3cs60303b

[b43] Pei S. & Chen H. M. The reduction of graphene oxide. Carbon 50, 3210–3228 (2010).

[b44] Stankovich S. . Synthesis of graphene-based nanosheets via chemical reduction of exfoliated graphite oxide. Carbon 45, 1558–1565 (2007).

[b45] McAllister M. J. . Single sheet functionalized graphene by oxidation and thermal expansion of graphite. Chem. Mater. 19, 4396–4404 (2007).

[b46] Wanga X., Liua B., Luc Q. & Qu Q. Graphene-based materials: Fabrication and application for adsorption in analytical chemistry. J. Chromatogr. A 1362, 1–15 (2014). 25160951 10.1016/j.chroma.2014.08.023

[b47] Yasunari T. J. . Cesium-137 deposition and contamination of Japanese soils due to the Fukushima nuclear accident. PNAS 108, 19530–19534 (2011). 22084074 10.1073/pnas.1112058108 PMC3241755

[b48] Romanchuk A. Y. . Graphene oxide for effective radionuclide removal. Phys. Chem. Chem. Phys. 15, 2321–2327 (2013). 23296256 10.1039/c2cp44593j

[b49] Pan H., Li J. & Feng Y. P. Carbon nanotubes for supercapacitor. Nanoscale Res. Lett. 5, 654–668 (2010). 20672061 10.1007/s11671-009-9508-2 PMC2894167

[b50] Yu G. . Enhancing the supercapacitor performance of graphene/MnO_2_ nanostructured electrodes by conductive wrapping. Nano Lett. 11, 4438–4442 (2011). 21942427 10.1021/nl2026635

[b51] Zhou W. . One-step synthesis of Ni_3_S_2_ nanorod@Ni(OH)_2_ nanosheet core–shell nanostructures on a three-dimensional graphene network for high-performance supercapacitors. Energy Environ. Sci. 6, 2216–2221 (2013).

[b52] Zhu G. . Highly conductive three-dimensional MnO_2_–carbon nanotube–graphene–Ni hybrid foam as a binder-free supercapacitor electrode. Nanoscale 6, 1079–1085 (2014). 24296659 10.1039/c3nr04495e

[b53] Dreyer D. R., Jia H. P. & Bielawski C. W. Graphene oxide: A convenient carbocatalyst for facilitating oxidation and hydration reactions. Angew. Chem. Int. Ed. 122, 6965–6968 (2010). 10.1002/anie.201002160 20602388

