# Supplementary material for: Tailoring the Oxygen Content of Graphite and Reduced Graphene Oxide for Specific Applications
Source: Sci Rep. 2016 Feb 25;6:21715. doi: 10.1038/srep21715 (PMC4766499; doi:10.1038/srep21715)
Supplement: Supplementary Information [file srep21715-s1.pdf]

Supporting Information

Tailoring the Oxygen Content of Graphite and Reduced Graphene Oxide for Specific Applications

Naoki Morimoto, Takuya Kubo and Yuta Nishina

1. Materials.

Graphite (SP-1) was purchased from BAY CARBON Inc. $KMnO_4$, H_2SO_4, 30% aq. H_2O_2, Cs_2CO_3, CsCl, hydrazine hydrate and methylene blue were purchased from Wako Pure Chemical Industries, Ltd. Benzylalcohol and 1,2-dichloroethane were purchased from Tokyo Chemical Industry Co. Acetylene black (DENKA black) was provided by DENKI KAGAKU KOGYO Inc. KF POLYMER 9130 was purchased from Kureha Co. All reagents were used directly without further purification.

2. General information.

X-ray diffraction (XRD) were measured by PANalytical Co. X' pert PRO using Cu Kα radiation (λ =1.541 Å) in the 2θ range of 2.0-75°. The operating tube current and voltage were 45 mA and 40 kV, respectively. The data was collected at the step size of 0.017° and the type of scan was continuous. XPS spectra were measured by SHIMADZU Kratos AXIS-ULTRA DLD with pass energy of 20 eV. Electrical conductivities were measured by using MITSUBISHI CHEMICAL ANALYTECH MCP-T610. The cyclic voltammograms of the second cycle were collected on Solartron 1287 electrochemical instrument at the scan rate 20 mVs^{-1}. The CV potential range was -0.8 to 0.1 V versus Hg / HgO in KOH electrolyte with a Pt foil as counter electrode. GC analyses were carried out with Shimadzu GC-2014 equipped with FID detector. Atomic absorption were made by using SHIMADZU AA-6300. Elemental analyses were performed by PERKINELMER 2400II. Freeze-dried of GO was made by using an ADVANTEC DRZ350WC. Raman spectra were measured by Horiba Jobin Yvon Inc. T-64000. UV-Vis spectra were obtained byJASCO V-550.

3. Preparation of several oxidation degree of GO by the oxidation of graphite (oGO).

Graphite (3.00 g) was stirred in 95% H_2SO_4 (75.0 mL). The required amount of $KMnO_4$ (0.75, 1.50, 2.25, 3.00, 3.75, 4.50, 5.25, 6.00, 7.50 and 9.00 g) was gradually added to the solution keeping the temperature <10 °C. The mixture was then stirred at 35 °C for 2 h. The resulting mixture was diluted by water (75.0 mL) under vigorous stirring. The suspension was further treated by adding 30% H_2O_2 solution (7.50 mL). The resulting graphite oxide suspension was purified by centrifugation with water. Several oGO were analyzed by CHN elemental analysis to evaluate the oxygen content. The result of elemental analysis is shown in Table S1.

Table S1. Elemental compositions of oGO.

Loading $KMnO_4$ (g)	C (w%)	H (w%)	N (w%)	O (w%)
0.75	81.2	0.9	0	17.9
1.50	75.0	0.9	0	24.1
2.25	69.9	1.0	0	29.1
3.00	64.4	1.2	0	34.4
3.75	62.0	1.3	0	36.7
4.50	59.0	1.2	0	39.8
5.25	53.7	1.8	0	44.5
6.00	49.3	1.7	0	49.0
7.50	43.1	1.7	0	55.2
9.00	39.3	1.9	0	58.8

4. Preparation of several oxidation degree of GO by the reduction of GO (rGO).

GO (2.00 g, O: 58.8 w%) which was synthesized by an above mentioned method using 6.0 g of $KMnO_4$ was dispersed in water (200 mL), then the required amount of hydrazine monohydrate (62.5, 125, 187.5, 250, 312.5, 375, 500, 750, 1000, 1500 and 2000 μL) was added before heating at 90 °C for 2 h. After cooling, the product was purified washed with water. Several rGO were analyzed by CHN elemental analysis to evaluate the oxygen content. The result of elemental analysis is shown in Table S2.

Table S2. Elemental compositions of rGO.

Loading hydrazine monohydrate (μL)	C (w%)	H (w%)	N (w%)	O (w%)
62.5	48.0	2.4	0.3	49.3
125	57.1	1.6	0.2	41.1
187.5	61.6	1.5	0.5	36.9
250	65.4	1.3	0.3	33.0
312.5	70.7	1.0	0.5	27.8
375	75.6	0.7	0.6	23.1
500	81.9	0.6	0.8	16.7
750	85.3	0.6	1.6	12.5
1000	84.8	0.5	2.1	12.6
1500	85.1	0.5	2.4	12.0
2000	85.2	0.5	2.5	11.8

5. XRD measurement of intermediate.

To confirm the homogeneous formation of graphite intercalated compound (GIC), XRD spectra were measured before treating with water.

Graphite (1.00 g) was stirred in 95% H_2SO_4 (25 mL). Small amount of $KMnO_4$ (0.10 g) was gradually added to the solution keeping the temperature <10 °C. The mixture was then stirred at 30 °C for 1 h. The mixture was centrifuged and XRD analysis was performed for the resulting precipitant.

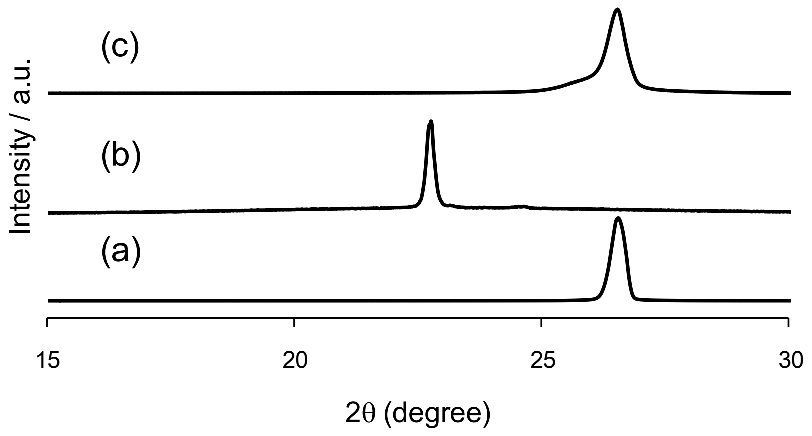

Figure S1. XRD spectra. (a) Graphite, (b) GIC, and (c) quenched GIC with water.

6. UV-vis spectroscopy.

Each sample was diluted to GO content of ca. 0.1 w% by ion exchanged water, then sonicated for 10 min before measurement.

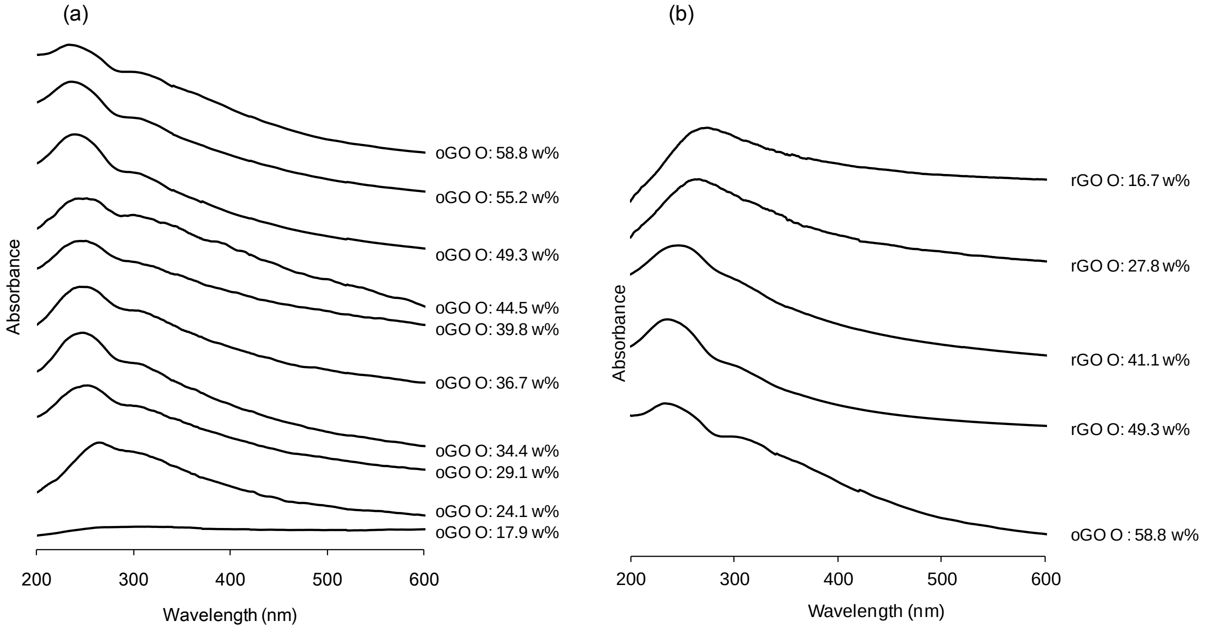

Figure S2. UV-vis spectra of GO with different oxygen content; (a) oGO and (b) rGO.

7. Raman spectroscopy.

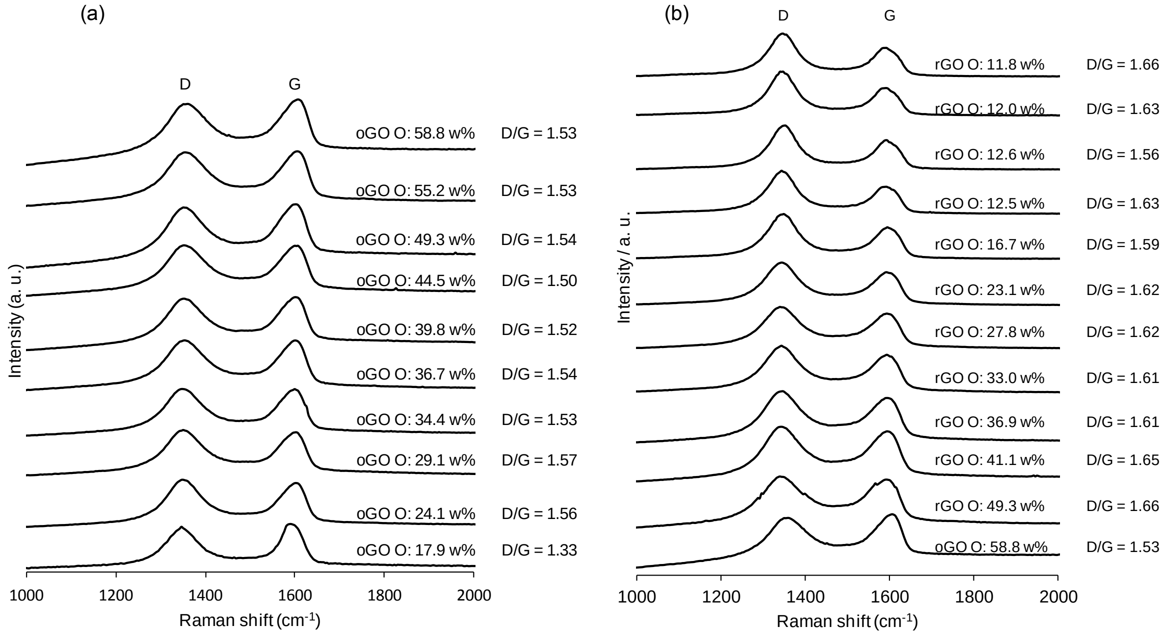

Figure S3. Raman spectra of GO with different oxygen content; (a) oGO and (b) rGO.

8. Oxygen functional group distribution analysis by waveform separation of XPS at C1s regions of oGO and rGO.

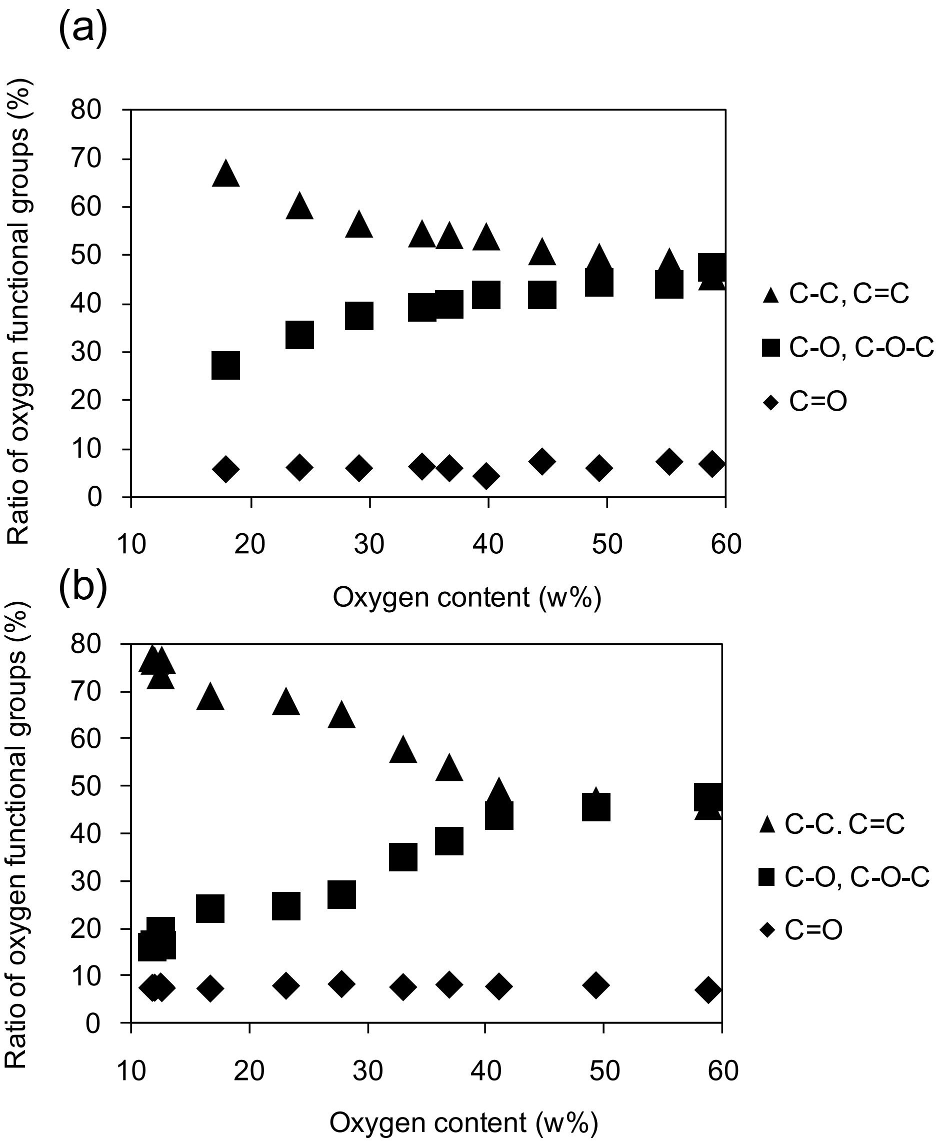

Figure S4. Analysis of oxygen functional group distribution determined by waveform separation of XPS at C1s regions; (a) oGO and (b) rGO.

9. Methylene blue (MB) absorption

To the solution of 23.3 w% aq. MB (66.7 mL) was added GO (10.0 mg). The mixture was sonicated (42 kHz, 130 W) for 90 min, then filtrated. The resulting solution was diluted to appropriate concentration and UV-Vis measurement was made at 298 nm

using stock solution of MB (0, 1.4, 2.1, 2.7, 4.0, 4.7, 5.4 w%) for calibration. The absorbed MB was calculated by subtracting the amount of MB which is not absorbed from total amount of MB.

10. Cs absorption

GO (20.0 mg) was added to the 20 mL of aqueous solution of 0.025 M of CsCl or 0.0125 M of Cs_2CO_3, then sonicated (42 kHz, 130 W) for 90 min. After the treatment, the reaction mixture was filtrated and the resulting solution was diluted to appropriate concentration, then atomic absorption of Cs was measured using stock solution of Cs (0, 0.2, 0.4, 1.0, 1.5, 2.0 μM) for calibration. The amount of absorbed Cs was calculated by subtracting the amount of Cs which is not absorbed from total amount of Cs.

11. Electrical conductivity measurement

Each sample was pelletized before the measurement using four-point probe. To investigate the electrical conductivity of GO, the average resistance was measured at 3 sampling points. The specific resistance was calculated according to

$\rho = RFL$

,where ρ is the specific resistance (Ωcm), R is the pellet thickness (cm), F is the correction coefficient which was determined from the distance between the probes attached with the instruments and L is the measured pellet resistance (Ω). The electrical conductivity (Scm^{-1}) was calculated reciprocal number of specific resistance.

12. Capacitance measurement

For the active electrode preparation, GO (30.0 mg), acetylene black (5.6 mg) and PVDf (2.0 mg) were mixed together in a mass ratio of 80 : 15 : 5, then N-methyl-2-pyrrolidine (13.1 mg) was added to the mixture. PVDf and acetylene black were used as the binder and conductive agent, respectively. Then the mixture was bound with Ni mesh and dried at 50 °C overnight in a vacuum oven. Active material (10.0 mg) was bound in the electrode. Each sample was directly used as electrodes in a three-electrode test cell using 1.0 M KOH aqueous solution as an electrolyte. Measurements were performed at the scan rate 20 mVs^{-1}. The gravimetric capacitance of the electrodes was calculated according to $C = \int idV/mv\Delta V$

,where C is the specific capacitance (Fg^{-1}), i is current (A), V is the potential (V), v is the scan rate (Vs^{-1}), and m is the mass of the active material (g). Calculation for $\int idV$ was made using cyclic voltammograms of the second cycle.

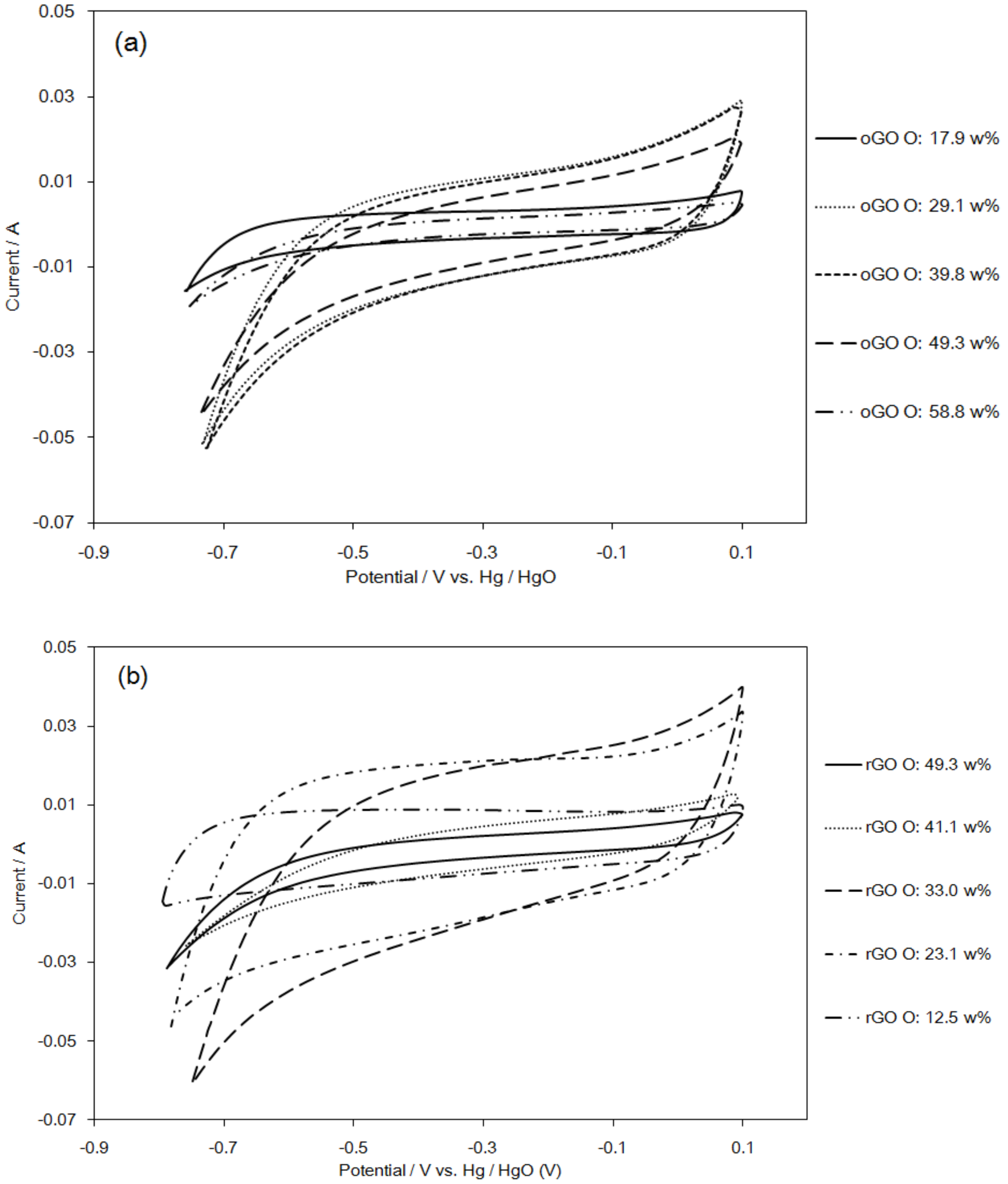

Figure S5. Cyclic voltammograms (20 mV/sec) of GO in 1.0 M KOH with different oxygen content; (a) oGO and (b) rGO.

13. Galvanostatic charge-discharge measurement

The potential range was chosen by taking into account the CV curves. The current density was fixed at 0.2 Ag^{-1} for direct performance comparison between the individual samples.

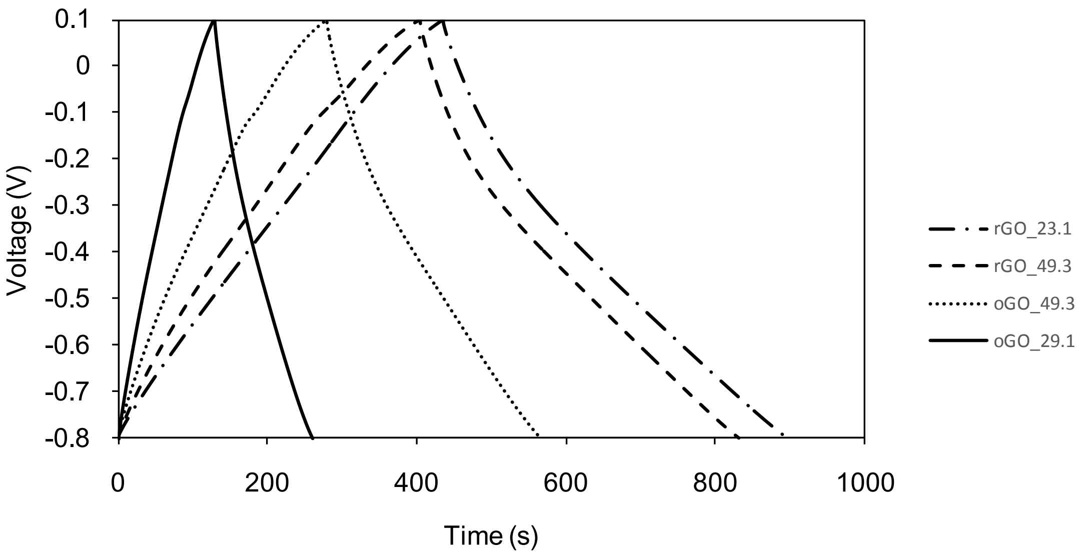

Figure S6. Galvano tatic charge/discharge curves of GO and rGO at 0.2 A g^{-1}.

14. Oxidation of benzylalcohol by using GO

To the solution of 1,2-dichloroethane (0.50 mL) was added GO(20.0 mg) in the presence of benzylalcohol (30.9 μL, 0.30 mmol) under Ar atomosphere and the mixture was stirred at 60 °C for 12 h. After the reaction, the reaction mixture was analyzed by gas chromatography using dodecane as an internal standard.
